# Effect of mutation at oxyanion hole residu (H110F) on activity of Lk4 lipase

**DOI:** 10.1016/j.btre.2021.e00590

**Published:** 2021-01-16

**Authors:** Ilma Fauziah Ma’ruf, Made Puspasari Widhiastuty, Suharti, Maelita Ramdani Moeis, Akhmaloka

**Affiliations:** a Biochemistry Research Group, Faculty of Mathematics and Natural Sciences, Institut Teknologi Bandung, Indonesia; b Genetic and Molecular Biotechnology Research Group, School of Life Sciences and Technology, Institut Teknologi Bandung, Indonesia; c Department of Chemistry, Faculty of Science and Computer, Universitas Pertamina, Indonesia

**Keywords:** Lipase, Oxyanion hole, Mutation, Catalytic activity

## Abstract

Mutant of lipase at oxyanion hole (H110 F) was constructed. The gene was highly expressed in *Eschericia coli* BL21 (DE3) and the recombinant protein was purified using Ni-NTA affinity chromatography. The activity of mutant enzyme was significantly increased compared to that the wild type. Further comparison showed that both of the enzymes exhibited same optimum pH and temperature, and showed highest lipolytic activity on pNP-decanoate (C10). The wild type appeared lost of activity on C14 and C16 substrates meanwhile the mutant still showed activity up to 20 %. In the presence of non polar organic solvent such as n-hexane, the wild type became inactive enzyme meanwhile the mutant still remained 50 % of its activity. The results suggested that mutation at oxyanion hole (H110 F) caused enzyme-substrate interaction change resulting on elevation of activity, better activity toward longer carbon chain substrate and improving the activity in the present of non polar organic solvent.

## Highlights

• Mutant of lipase (Lk4) gene was constructed through PCR site directed mutagenesis.
• The mutant Lk4 (H110 F) caused increasing of catalytic activity. The enzyme showed same optimum pH and temperature compared to that the wild tpe.
• The mutant showed better activty toward longer carbon chain substrate and improving the activity on the presence of non polar organic solvent.

## 1 Introduction

Lipase is an enzyme catalyzing carboxyl ester bonds formation or its hydrolysis. In presence of water the enzyme catalyze carboxylate ester bonds hydrolysis to produce free fatty acids and organic alcohols. Meanwhile in low water environment, the enzyme shows esterification or transesterification activity such as alcoholysis, acidolysis, aminolysis and interesterification [1,2]. Lipase shows important role in many industrial applications such as additive in biodetergent, pulp and paper industries, leather and food industries, fine chemicals synthesis, biofuels and biodegradable polymers productions [[3], [4], [5]].

Lipase shows similar structure with other hydrolases (esterase, protease, dehalogenase etc) called α/β hydrolase fold that contain eight β pleated sheets sorrounded by α helixes. The enzyme consists some important structural features such as catalytic triad, oxyanion hole, and lid. Catalytic triad of lipase consists of one nucleophilic (serine), one catalytic acid (aspartic or glutamic residue) and one of histidine residues [6,7]. The enzyme also showed a pocket called oxyanion hole that has role in stabilization of negative oxygen carbonyl group of substrate during intermediate formation. Oxyanion hole consists two residues, the first residue is located between β3-strand and αA-helix. The second residue is located at pentapeptide motive on the C-terminus of catalytic residue of serine [8,9]. Highly conserved pentapeptide motive shows pattern G-X-S-A-G, X refers to second oxyanion residue and S refers to serine as nucleophilic residue [10]. Lid has a role of covering active site of the enzyme in the absence of lipid-water interface. While in the presence of interface, lid will open and the substrate interact to the catalytic site [11,12].

Catalytic activity of lipase might be improved by mutation on the catalytic pocket of oxyanion hole [9,13]. There are a few reports showed that mutation on oxyanion hole residues affect features of various lipase [[14], [15], [16], [17], [18]]. Thermostable lipase from *G. zalihac* contains two oxyanion hole residues, Q114 and F16. Mutation on Q114 L resulted on mutant enzyme with better properties such as increasing on optimum temperature, stability in organic solvent and surfactant resistency [16]. Mutation at oxyanion hole (F146 L) of *Yarrowia lipolytica* lipase caused on changing in substrate binding and catalytic efficiency [17]. F17S mutation on *B. thermocatenolatus* lipase exhibited higher activity in the presence of organic solvent [18].

Thermostable lipase were currently highly explored since the enzyme might increase productivity due to increasing reaction rate at high temperature. Exploration of thermostable lipases were extensively carried out [19]. Various lipases were isolated from local strains and traditional compost [[20], [21], [22], [23], [24]]. One of the lipase gene namely *LK_ITB5a* (*LK4*) was previously isolated directly from compost using metagenomic approach at thermogenic phase. The gene consists 936 base pairs coding for 311 amino acids. The lipase has 99 % similarity with lipase from *Pseudomonas stutzeri* AID66451.1. Nucleotide sequence of *LK4* was deposited into GeneBank with accession number of KP204886 [21]. The lipase has three catalytic triads (Ser109, Asp255 and His277) and two oxyanion hole (Met43 and His110) residues. This paper reported the first mutation in one of oxyanion hole (H110 F) on *Pseudomonas stutzeri* Lk4 lipase that significantly increasing (4 times) the activity of the enzyme.

## 2 Methods

### 2.1 Computational method

The 3D structure of proteins was constructed by using *SWISS MODEL* program (https://swissmodel.expasy.org/interactive) [25]. Lk4 has closest identity with PDB ID 1ex9, which further used as template for 3D structure protein construction. The proteins was visualized by using *UCSF Chimera* [26]. Ligand (pNPD) was created and energy-minimized using *Marvin Sketch*(https://chemaxon.com/products/marvin). Molecular Docking was performed using *Autodock Vina* [27]. Gridbox was set as follows (center_x = 0.055, center_y =-2.765, center_z = 45.894, size_x = 12, size_y = 14, size_z = 10). *Ligplot+* used to visualized ligand-proteins coordinate after docking process [28]. Interaction between ligand and Lk4/Lk4H110 F also visualized using *PLIP* (https://projects.biotec.tu-dresden.de/plip-web/plip) [29]. Standalone *Voronoia* software was used to estimate total cavities of proteins [30].

### 2.2 Construction of mutant

The wild type of lipase gene from pJET-*Lk4* plasmid was amplified using Feksp and Reksp primers. The mutant was constructed by PCR-site directed mutagenesis using primers namely Feksp (5’-CAACATATGAACAAGAACAAAACCTTGCTCGCC-3’), Reksp (5’-AAAGTCGACGAGCCCCGCGTTCTT-3’) [31] and RF3 (5’-GTCGGTCCGCCGAAGCTGTGGCCG-3’). The first PCR process using Feksp and RF3 primers produced 348 bp amplicon. The amplicon was used as mega primer paired with Reksp primer for the second PCR process to get whole mutant gene. Following confirmation of the mutation, the mutant gene was inserted on the pET-30a(+) resulting on recombinant plasmid pET-*LK4*H110 F. The recombinant plasmid was used to transform *E. coli* BL21 (DE3).

### 2.3 Heterologous expression of protein

Transformant cells were inoculated in LB broth contain kanamycin 50 μg/mL and incubated at 37 ⁰C, 150 rpm, overnight. The culture was inoculated to fresh LB broth and incubated at 37 ⁰C, 150 rpm until OD_600_ was 0.6−0.8. IPTG was added until final concentration 1 mM. Protein expression was performed at 37 ⁰C, 150 rpm for 4 h. Pellet cell was harvested by centrifugation at 4500 *g* for 10 min. The protein was extracted using SDS-heating method as follows. The pellet was added with lysis buffer (sodium phosphate buffer 0.05 M pH 8 contain SDS 0.1 % v/v) and shaked with BenchRocker 2D for 30 min at room temperature. Crude extract obtained by lyse the cell suspension at 60 ⁰C for 10 min. Supernatant was separated from cell debris by centrifugation at 12,000 *g* for 30 min. Supernatant was incubated for 30 min at 60 ⁰C to remove proteases

### 2.4 Purification of recombinant protein using IMAC Ni-NTA

Purification was performed by gravity flow method at room temperature. 4 mL Ni-NTA agarose suspension was filled into a column. The column was then washed with 3 × 10 mL miliQ water. Binding buffer (sodium phosphate buffer 0.05 M, pH 8, NaCl 100 mM and Triton X 0.1 % v / v) of 3 × 15 mL were passed into the column. A protein solution (∼ 45 mL from ∼1.5 g cell) was passed into the column. The resin was washed with a buffer (sodium phosphate buffer 0.05 M pH 8, NaCl 100 mM) of 2 × 40 mL. The bound proteins were eluted using elution buffer gradually containing 10 mM imidazole (sodium phosphate buffer 0.05 M pH 8, NaCl 300 mM, and imidazole 10 mM), 10 mL elution buffer 80 mM imidazole (sodium phosphate buffer 0.05 M pH 8, NaCl 300 mM, imidazole 80 mM) and 4 mL elution buffer containing imidazole 150 mM (sodium phosphate buffer 0.05 M pH 8, NaCl 300 mM, imidazole 150 mM). Eluted proteins with 80 mM imidazole was diafiltrated using Amicon Ultra Centrifugal Filter (with 10 kDa cut off membrane) until imidazol concentration was ∼ 0.002 mM.

### 2.5 Lipolytic assay and characterization

Lipase activity was determined by modified colorimetric method using *para*-nitrophenyl fatty acids as substrate [24]. 300 μL protein solution was added to 900 μL substrate mixture (sodium phosphate buffer 0.05 M pH 8: Ethanol: substrate solution 10 mM = 95:4:1). Reaction mixture incubated at 50 ⁰C pH 8 for 15 min. Reaction process stopped by incubating the mixture on ice. One Unit of lipase is defined as the amount of enzyme releasing 1 μmol pNP /min. Specific activity defined as Unit/mg proteins.

To probe substrate specificity assay C10-C16 analogue substrate (p-nitrophenyl Decanoate/ pNPD, p-nitrophenyl Laurate/ pNPL, p-nitrophenyl Myristate/ pNPM and p-nitrophenyl Palmitate/ pNPP) were used for the reaction. Reaction mixture was incubated in standard reaction as described before. To probe optimum temperature, the assay was performed using pNPD as substrate at temperature range at 30−80 °C, pH 8. Reaction was performed in standard reaction described before. To measure optimum pH, the assay was performed using pNPD as substrate and reaction mixture incubated at various pH (sodium phosphate buffer 0.05 M for pH 6–8 and glycine-NaOH buffer for pH 9–11) at 15 min. To monitor lipase activity in the pesence of organic solvents, methanol, ethanol, isopropanol, aceton, acetonitrile, chloroform and n-hexane at 3% concentration were used. Assay was performed at 40 ⁰C, pH 9 and use standard reaction as described before. To probe stability of enzyme at 40 ⁰C, enzyme was firstly incubated on that condition for 0−240 min. Following incubation, the assay was performed at 40 ⁰C, pH 9 and use standard reaction as described before.

## 3 Result and discussion

### 3.1 Lipolytic activity of Lk4 H1104 mutant

Histidine at position 110 of Lk4 was confirmed as one of oxyanion hole [21]. The residue was replaced into phenylalanine through PCR directed mutagenesis. Mutation of H to F was carried out since the amino acids structurally similar, both residues contain bulky side chain, however, histidine contains free electron pair as nucleophile while phenylalanine lack of nucleophylic property. The gene was inserted on the expression vector pET30a(+) fused by His-tag. Following expression of the gene, the protein was purified by IMAC NiNTA. Both proteins (wild type and mutant) exhibited maximum lipolytic (ester hydrolysis) activity on para-nitrophenyl decanoate (pNPD) as substrate. The mutant protein showed higher specific activity (0.0508 U/mg) compared to that wild type (0.0115 U/mg) (Table 1). Increasing the activity of mutant enzyme up to 4 times was not surprising since substitution of residue on oxyanion hole might change conformation of catalytic pocket. Oxyanion hole residue of lipase is conformationally close to catalytic residues. Substitution on the residue might change the interaction of catalytic residues with substrate as shown on mutation at I12 F *Bacillus substilis* lipase [16]. In case of Lk4 H110 F mutant, the activity of the enzyme significantly increase, might due to change in local conformation of the enzyme especially on surrounding of the catalytic pocket since phenylalanine carrying large hydrophobic aromatic side group. As consequence, the substitution might change orientation of –NH group on the oxyanion hole so that increasing stabilization of transition state and hence increasing activity of the enzyme [32].

**Table 1:** Purification of wild type and mutant lipases using IMAC NiNTA. CE: crude extract, P: purified enzyme.

		Total Activity (U)	Specific Activity (U/mg)	Yield (%)	Purification fold (x)
Lk4	CE	0,1172	0,0017	100	1
	P	0,0067	0,0115	6	6,9339
Lk4 H110F	CE	0,0954	0,0023	100	1
	P	0,0563	0,0508	59	22,4010

**Fig. 1:**
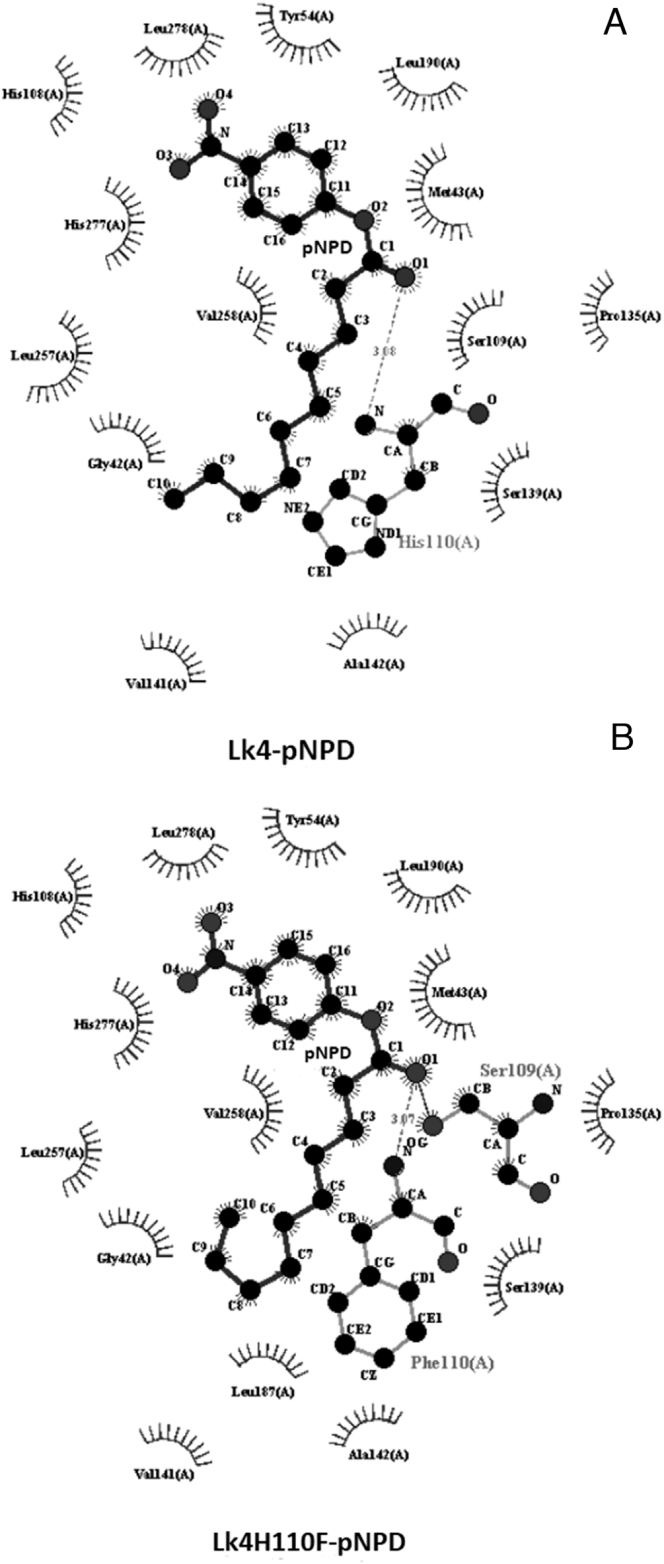
Enzyme-substrate interaction coordinate. A: Lk4-pNPD, B: Lk4H110F-pNPD.

To further probe the molecular mechanism of the above phenomenon, molecular docking assay was subjected to enzymes using pNPD as substrate. The result showed that both enzyme (Lk4 and Lk4 H110 F) exhibited same estimated binding energy with value of –6.7 kcal/mol. On the other hand, protein-ligand coordinate plot using *Ligplot^+^* program showed that hydrogen bond between amino (-NH) residue of oxyanone hole and oxygen (O) residue at C1 of pNPD is shorter in the mutant (3.07 Å) compared to that in the wild type (3.08 Å). In mutant enzyme the substrate was seen closer to viscinity to oxygen (O) residue at gamma position of nucleophilic residue (S109) (Fig. 1). In which serine will form covalent bond with substrate during intermediate formation [33]. Further characterization on the substrate-protein interaction distance using *PLIP* program revealed that the distance of hydrogen bond involving S109 and salt bridges involving H277 between enzyme and substrate were shorter in mutant compared that the wild type (Table 2). Both data are in agreement that substitution on histidine to phenylalanine at Lk4 might change on local conformation of catalytic pocket and hence increasing activity of the enzyme.

**Table 2:** Enzyme-substrate interaction distance created using *PLIP*.

	Lk4		Lk4 H110 F	
	Residu involved	Distance (Ǻ)	Residu involved	Distance (Ǻ)
Hydrogen bond	S109	2.74	S109	2.6
	H110	3.08	F110	3.07
Salt bridge	H277	5.1	H277	4.96

### 3.2 Substrate preference

**Fig. 2:**
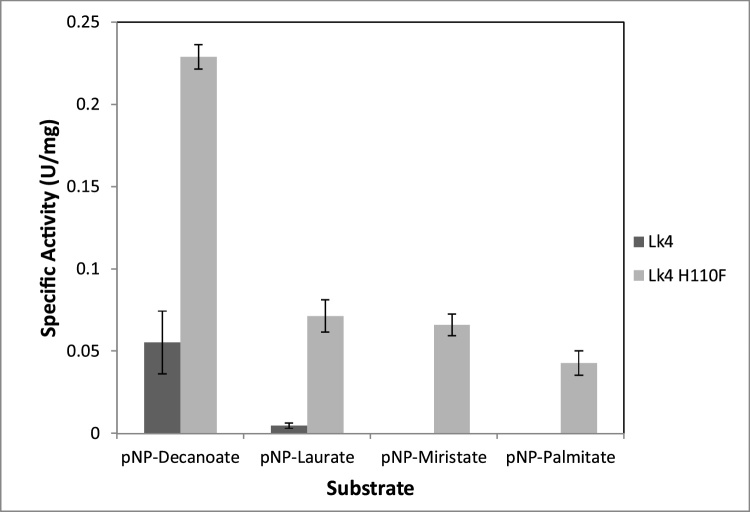
Activity of Lk4 and Lk4H110 F on various different substrate. The assay was carried out at 50 °C, pH9.

Most of lipases are specific to a particular type of substrate especially on carbon length of substrate [34]. A few lipases are also specific to substrate containing double bonds on particular position [15]. Mutation at D94 S on oxyanion hole of *Rhizopus oryzae* lipase (*RO*L) caused shifting toward shorter carbon chain substrate [14]. Geometry and hydrophobicity of catalytic pocket determine chain length specificity of lipase. Although geometry at catalytic triad is highly conserve, high variability of the catalytic pocket cause different specificity of lipase for particular substrate [24]. It is also determined by hydrophobic interaction between catalytic pocket residues and ligand’s acyl chain [35]. Mutation of Lk4 at H110 F seem slightly alter specificity of the enzyme on preferencies of the substrates (Fig. 2). Both enzymes showed highest activity on pNPD as substrate. Moreover the wild type exhibited lost of activity on C14 and C16 carbon chain substrate meanwhile the activity of mutant still remained up to 20 %.

### 3.3 Effect of organic solvents on enzyme activity

**Fig. 3:**
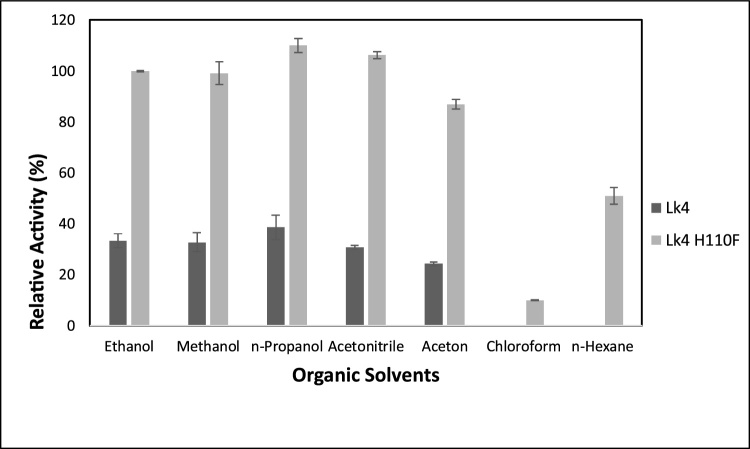
Activity of Lk4 and Lk4H110 F various organic solvents. The assay was carried out using pNPD as substrate at 40 °C, pH9.

In the presence of organic solvent, most of enzymes are easily denatured and inactivated. However, some lipases still remain functional since the enzymes have rigid conformation and active under two phase systems [36]. Lk4 exhibited some variation activities in polar organic solvent. However, the enzyme was completely lost of the activity in the presence of nonpolar organic solvent such as chloroform or n-hexane (Fig. 3). In contrast, the mutant enzyme still showed 50 % activity in the presence of n-hexane compared to that the control (ethanol). Improving activity of the enzyme in the present of organic solvent was reported on F17 S mutant of *B. thermocatenolatus* lipase [18]. Our previous report showed that lipase from *Pseudoxantomonas* sp. still exhibited an activity in the presence of methanol up to 30 % concentration [37]. Furthermore, lipase from *Geobacillus thermoleovorans* showed tolerance to various polar solvents and loss of activity in the presence of n-hexane and chloroform [38]. In conclusion, hydrolytic activity of lipases was higher in the presence of water-miscible solvent compared to that on water immiscible solvent. Remaining 50 % activity of the mutant (Lk4 H1104) in the presence of n-hexane might be due to substitution of histidine to phenylalanine changed on polarity of oxyanion hole influencing on conformation of catalytic pocket. As consequence the catalytic activity of the enzyme was still retained on the presence of nonpolar organic solvent.

### 3.4 Effect of temperature and pH on enzyme activity

**Fig. 4:**
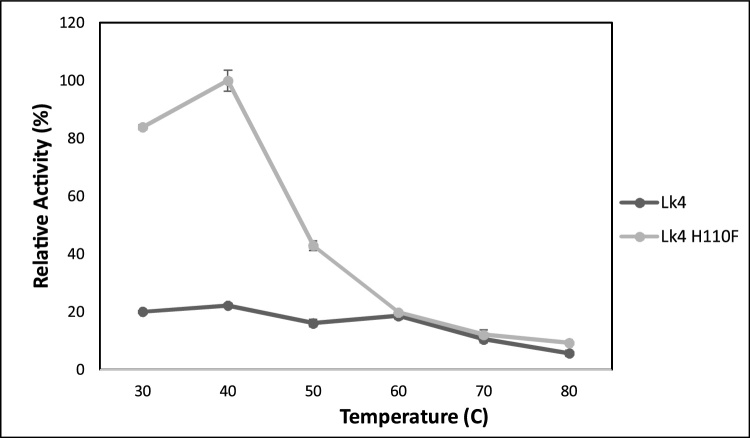
Activity of Lk4 and Lk4H110 F on various temperature. The assay was performed using pNPD as substrate, pH8.

Lipases from various *Pseudomonas* species have been studied extensively. Each lipase exhibited optimum activity on diverse temperature: *P. fluorescens* lipase (40 °C) [39], *Pseudomonas* sp. f-B-24 lipase (40 °C) [40], *P. aeruginosa* KM110 lipase (45 °C) [41], *Pseudomonas* sp. AMS3 lipase (50 °C) [42], *P. aeruginosa* SRT9 lipase (55 °C) [43]. To probe the effect of the temperature on the activity of enzymes, the mutant and wild type enzymes were assayed at variation temperature from 30 up to 80 °C. Both enzymes showed optimum temperature at 40 °C. The activity of Lk4 showed insignificant difference at temperature range from 40−60 °C (Fig. 4). Surprisingly the activity of mutant (Lk4 H110 F) enzyme was dramatically decreased at temperature above 40 °C. Substitution from histidine to phenylalanine might changed electrostatic force of the residue from negative charge (H) to nonpolar (F) residue and increased steric hindrance on the oxyanion hole. For more detail to slightly evaluate substitution from H to F on steric hindrance effect, total cavities of both enzymes were determined through *Voronoia* program. The result showed that total cavities of mutant was higher compared to that the wild type (Table 3). It is suggesting that an alleviation temperature in the mutant influences on conformational stability of mutant enzyme and hence destabilization of intermediate enzyme complex.

**Table 3:** Total cavities prediction of wild type and mutant lipases.

	Total Cavities (Voronoia)
Lk4	18
Lk4H110F	22

**Fig. 5:**
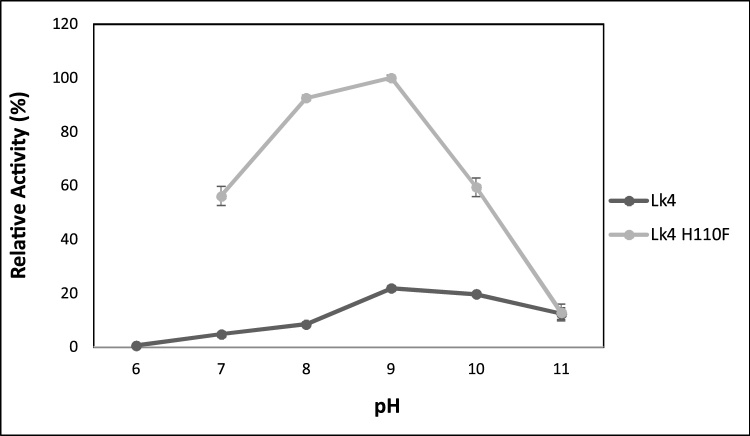
Activity of Lk4 and Lk4H110 F on various pH. The assay was performed using using pNPD as substrate at 40 °C.

Most of lipase showed optimum activity at neutral to slight alkaline pH [44,45]. Lk4 showed an alkaline lipase with optimum activity at pH 9 (Fig. 5). Most of *Pseudomonas* lipases were reported exhibited optimum pH at pH 7–8, only a few of them showed optimum pH at 9 [42]. Optimum pH is determined by some factors such as composition of amino acids (pI), and arrangement of titratable groups in protein structure [46]. Isoelectric point of Lk4 and Lk4 H110 F are 6.13 and 6.05 respectively. Meanwhile both enzymes showed optimum activity at pH 9. It seems that the enzymes should be negatively charge to perform the activity. At pH 11 the enzyme still remained 75 % activity. Mutant enzyme also exhibited same optimum pH compared to that the wild type, however at pH above 9, the activity of the mutant was dramatically decrease. At pH 11, activity of the mutant showed less than 20 %. Increasing on pH gives addition of hydroxyl ion concentration. It seemed that additional of hydroxyl ions did not influence on the conformation and interaction between catalytic residues with substrate on Lk4 but did significantly influence on the mutant. The significant influence of the effect of additional hydroxyl ion to the mutant might due to less of pI value of the mutant and hence increasing of negative charge of the protein.

### 3.5 Thermostability of enzymes

**Fig. 6:**
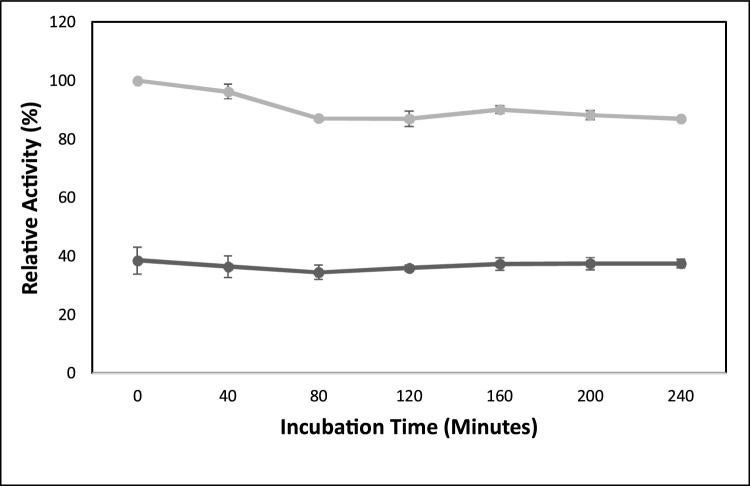
Activity of Lk4 and Lk4H110 F after incubation at 40 °C, pH9. The assay was performed using pNPD as substrate.

The optimum temperature of Lk4 and Lk4 H110 F were at 40 °C, moreover the activity of Lk4 remained similar up to temperature at 60 °C (Fig. 5). In contrast, the mutant enzyme was lost of the activity up to more than 75 % at 60 °C. For further analysis concerning thermostability of the enzymes, both enzymes were assay by incubation up to 4 h at the optimum temperature (40 °C) and pH 9 (Fig. 6). After 4 h incubation, the wild type enzyme was shown relatively stable, remaining activity of 97 %. In another hand, after 4 h incubation remained activity of the mutant was 87 %. The higher loss of mutant activity compared to that the wild type was in line with the predicted total cavity of the molecule. Higher total cavity resulted on more water molecules buried in it and caused destabilization of Van der Walls and hydrophobic interaction among amino acid residues [47]. It seems that the role of oxyanion hole residues do not only to bind substrate but also regulate conformational stability of the enzyme. Since oxyanion holes residues lied inside the catalytic pocket, substitution on oxyanion residues might change the protein compactness and hence changes stability of protein [16]. In the case of the mutant enzyme showing higher activity but less stability is probably due to related on structural stability of the mutant. In many cases, enhancing protein flexibility leading on increasing protein activity but less stability [48]. Mutation at F17 S involved on oxyanion formation in chimeric lipase from *B. thermocatenulatus* also show increasing activity but decreasing thermostability [18].

From all of the data suggesting that mutation on H110 F on Lk4 showed changing on substrate-enzyme interaction resulting on increasing activity, shifting toward longer carbon chain substrate, improving the activity in the present of nonpolar organic solvent, however slightly decreasing in thermal stability of the enzyme.

## 4 Conclusion

Expression vector of pET-*LK4* and pET-*LK4*H110 F were successfully constructed and expressed in *E. coli* BL21 (DE3). Crude extract of both proteins exhibited lipolytic activity on pNPD as substrate. The purified enzymes showed same optimum pH and temperature, substrate preference, and maintained the activities in presence of polar or semipolar organic solvent. The activity of mutant enzyme showed much higher catalytic activity compared to that the wild type. In addition, the wild type enzyme lost of its activity in nonpolar organic solvent and long carbon chain (C14 and C16) substrates meanwhile the mutant still exhibited the activity.

## Author contribution statement

All authors listed have made a substantial, direct and intellectual contribution to the work and approved it for publication.

## Additional information

No additional information is available for this paper.

## Declaration of Competing Interest

The authors report no declarations of interest.
